# Model-based estimation of transmissibility and reinfection of SARS-CoV-2 P.1 variant

**DOI:** 10.1038/s43856-021-00048-6

**Published:** 2021-11-15

**Authors:** Renato Mendes Coutinho, Flavia Maria Darcie Marquitti, Leonardo Souto Ferreira, Marcelo Eduardo Borges, Rafael Lopes Paixão da Silva, Otavio Canton, Tatiana P. Portella, Silas Poloni, Caroline Franco, Mateusz M. Plucinski, Fernanda C. Lessa, Antônio Augusto Moura da Silva, Roberto Andre Kraenkel, Maria Amélia de Sousa Mascena Veras, Paulo Inácio Prado

**Affiliations:** 1 Universidade Federal do ABC, Santo André, Brazil; 2 Observatório COVID-19 BR, São Paulo, Brazil; 3 Universidade Estadual de Campinas, Campinas, Brazil; 4 Universidade Estadual Paulista, São Paulo, Brazil; 5 Vigilância Epidemiológica, Secretaria de Saúde de Florianópolis, Florianópolis, Brazil; 6 Universidade de São Paulo, São Paulo, Brazil; 7 Centers for Disease Control and Prevention, Atlanta, GA USA; 8 Universidade Federal do Maranhão, São Luís, Brazil; 9 Faculdade de Ciências Médicas da Santa Casa de São Paulo, São Paulo, Brazil

**Keywords:** Viral infection, Dynamical systems

## Abstract

**Background:**

The SARS-CoV-2 variant of concern (VOC) P.1 (Gamma variant) emerged in the Amazonas State, Brazil, in November 2020. The epidemiological consequences of its mutations have not been widely studied, despite detection of P.1 in 36 countries, with local transmission in at least 5 countries. A range of mutations are seen in P.1, ten of them in the spike protein. It shares mutations with VOCs previously detected in the United Kingdom (B.1.1.7, Alpha variant) and South Africa (B.1.351, Beta variant).

**Methods:**

We estimated the transmissibility and reinfection of P.1 using a model-based approach, fitting data from the national health surveillance of hospitalized individuals and frequency of the P.1 variant in Manaus from December-2020 to February-2021.

**Results:**

Here we estimate that the new variant is about 2.6 times more transmissible (95% Confidence Interval: 2.4–2.8) than previous circulating variant(s). Manaus already had a high prevalence of individuals previously affected by the SARS-CoV-2 virus and our fitted model attributed 28% of Manaus cases in the period to reinfections by P.1, confirming the importance of reinfection by this variant. This value is in line with estimates from blood donors samples in Manaus city.

**Conclusions:**

Our estimates rank P.1 as one of the most transmissible among the SARS-CoV-2 VOCs currently identified, and potentially as transmissible as the posteriorly detected VOC B.1.617.2 (Delta variant), posing a serious threat and requiring measures to control its global spread.

## Plain language summary

SARS-CoV-2, the virus that causes COVID-19, can mutate into variants which might have different characteristics such as increased ability to spread. The P.1 variant, also known as Gamma, was first identified in Manaus, Brazil. Here, we use mathematical and statistical methods to model the spread of P.1 in Manaus and to determine the potential for people getting re-infected with P.1, comparing it to previous circulating variants in the region. We estimate that P.1 spreads 2.6 times more easily than these other variants and that nearly a third of cases over the period studied were reinfections with P.1. These findings help us to understand whether P.1 is likely to be more difficult to control than other variants and what sort of measures might be needed.

## Abstract (web summary)

Coutinho et al. use genomic and epidemiological data from Manaus, Brazil, to estimate the transmissibility and potential for reinfection with the SARS-CoV-2 variant-of-concern P.1 (gamma). Using mathematical modelling, the authors estimate that P.1 is 2.6 times more transmissible than previous SARS-CoV-2 variants circulating in Manaus and underlies a large proportion of reinfections in Manaus.

## Introduction

The Japanese National Institute of Infectious Diseases identified the new P.1 SARS-CoV-2 variant from travelers returning from Amazonas State, Brazil, on 6-January-2021^1^. P.1 was eventually reported in Manaus city (Amazonas state capital), on 11 January-2021^2^. Later, it was identified in samples collected since 6-Dec-2020 from Manaus^3^. According to phylogenetic studies, P.1 likely emerged in the Amazonas state in early^3^ or late^4^ November 2020. This variant shares mutations with other variants of concern (VOCs) previously detected in the United Kingdom and South Africa (B.1.1.7 and B.1.351, respectively)^2^. Mutations of these two other variants are associated with greater transmissibility and immune evasion^5,6^, which confer them the status of variant of concern. However, information, data, and analyses on the epidemiology of P.1 are still incipient.

**Fig. 1:**
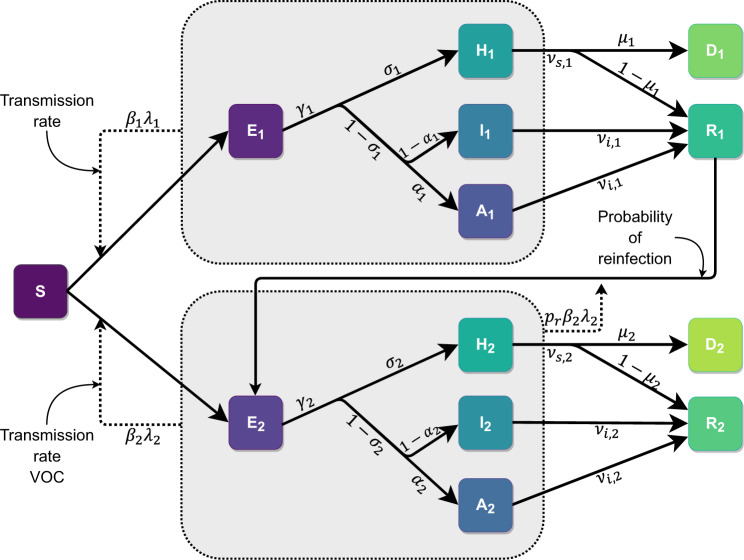
Diagram of the extended deterministic compartmental model (SEAIHRD). The model compartments and the respective connections between them are summarized in this diagram, and they are named as S: Susceptible, E: Exposed (pre-symptomatic), H: Hospitalized (severe infected individuals), I: Infected (symptomatic individuals, not hospitalized), A: Asymptomatic. D: Deceased, R: Recovered. Compartments are subdivided into three age categories, not represented here for simplicity. Compartments with subindex 1 represent the wild-type variant, subindex 2 refers to the VOC P.1. Continuous lines represent flux between each compartment; dashed lines, infection pathways. Small arrows indicate force of reinfection and transmissibility. *λ* = force of infection. *β* = relative transmission rate. *p*_*r*_ = relative force of reinfection. *γ* = average time between being infectious and presenting symptoms. *σ* = proportion of severe cases that require hospitalization. *α* = proportion of asymptomatic cases. *ν*_*s*_ = average time between being infectious and recovering for severe cases. *ν*_*i*_ = average time between being infectious and recovering for mild/asymptomatic cases. *μ* = in-hospital mortality ratio.

The Coronavirus disease 2019 (COVID-19) outbreak in Manaus (April–May 2020) was followed by a period of high but stable incidence, after which the proportion of individuals who were infected by the SARS-CoV2 virus may have reached 42%^7^ to 76%^8^ by November 2020. From December 2020 to February 2021 the city was devastated by a new outbreak that caused a collapse in the already fragile health system^9^, with shortages of oxygen supply^10^, while the frequency of P.1 increased sharply from 0% in November 2020 to 73% in January 2021^4^. The pathogenicity of P.1 variant is still unknown, although recent studies point to increased viral load in individuals infected with the new variant^4^, suggesting it could be higher than the one from previous circulating strain. We analyzed Brazilian national health surveillance data on COVID-19 hospitalizations and the frequency of P.1 among sequences from residents of Manaus city using a model-based approach (an extended SEIR compartmental model—see Fig. 1) to estimate the **r**elative transmissibility in comparison to the previous local variant(s), and **r**elative force of reinfection of the P.1 variant, i.e. the ratio between the force of infection by P.1 on previously infected individuals (reinfections) and the force of infection by P.1 on susceptible ones (new infections). We estimate that the P.1 variant is about 2.6 times more transmissible than previous circulating variant(s), and 28% of Manaus cases in the period were due to reinfections.

## Methods

In order to estimate key parameters of the variant of concern (VOC) P.1, we developed a model and fitted it to time-series data of number of hospitalized cases and frequency of the P.1 variation. The fitting approach used here can be applied to other regions where as soon as data on number of cases and frequency of a new variant are available. It primarily requires surveillance data to determine proper model initial conditions. In Brazil, these are the hospitalized cases data. Stratification by age allows the model to also consider the different death rates, asymptomatic and hospitalized proportions of each age class, important features for SARS-CoV-2. Contact levels between different age classes, which may vary from one place to another, can also be considered. For special cases in which information such as contact between ages classes and age distribution are not available (or even unnecessary for some other disease), the model can be easily simplified. In this sense, the method proposed here demands low-detailed data and relies on the structure of a simple compartmental model to measure quantities of interest, such as transmissibility and relative force of reinfection. More information about the methodology here applied is available in the Supplementary Information (SI) in sections 1–3.

### Model

A deterministic compartmental model (Fig. 1) was developed to model the infection of Susceptible individuals moving to the Exposed (pre-symptomatic) compartment, which can progress to three alternative compartments: Hospitalized (severely ill), Infected (symptomatic but non-hospitalized), and Asymptomatic. Eventually, individuals move to Recovered or Deceased. Two variants are considered: 1-non-P.1 (“wild-type”) and 2-new/P.1. The latter is assumed to infect Recovered individuals previously infected by the wild-type, and no reinfections of wild-type due to waning immunity occur. Compartments were stratified into three age categories: young (<20 years old), adult (≥20 and <60 years old) and elderly (≥60 years old), with different rates for outcomes. The key parameters of relative transmissibility and relative force of reinfection were estimated by a maximum likelihood fitting to the weekly number of new hospitalizations and to genomic surveillance data. Three additional model parameters with unknown values were also estimated. The remaining parameters (24 out of 29) were fixed, using current values from the literature. Sensitivity to different pathogenicity of the P.1 variant was explored by repeating the fit assuming IHR as a free parameter (SA1). The sensitivity to the period analysed was also explored by another fit excluding the health system collapse period (SA2) (see below. Further model details about the model are available in the Supplementary Methods (Section 1 of the SI).

### Dataset

We used the Brazilian epidemiological syndrome surveillance system for influenza, SIVEP-Gripe (https://opendatasus.saude.gov.br), to track COVID-19 hospitalized cases. All hospitalized patients with Severe Acute Respiratory Illness (SARI) are reported to SIVEP-Gripe with symptom onset date and SARS-CoV-2 test results. SIVEP-Gripe, due to its universal coverage and mandatory notification of SARI cases, has an homogeneous testing effort to diagnose SARS-CoV-2 infections, and is currently the best source for Brazilian data at the national level. As the data are publicly available by the Brazilian National Health System, no ethical approval was needed to perform the analysis, according the the National Ethical Commission (CONEP) of the National Health Council, Resolution Number 510 of April 7, 2016. Hospitalization data provides the most accurate basis to infer incidence in Manaus, because mild cases are vastly under-reported and testing capacity fluctuates, while mortality data are harder to relate to total number of cases, since the city’s health system endured a prolonged stress even before the collapse, with large variation in the in-hospital fatality rate over time^11^. Time-series of frequency of sequenced genomes identified as P.1 in Manaus were extracted from published datasets^3,12^. Because of the uncertainty of the mortality rate, hospitalization data are more reliable to monitor the Brazilian situation. However, hospitalization suffers an important dampening when the health system collapses, presenting a false spreading control precisely because of the overload on the health services, which cannot admit more patients than their capacity. We take this into account making a sensitivity analysis (SA2—see below). Likewise, when the health system is overwhelmed, the IHR can be higher (as it can be only a trait of the P.1 variant), leading to greater mortality rates than the parameters used in this work. For this reason, we also taken the differing IHR into account for P.1 using a sensitivity analysis (SA1—see below). Data and code are available in the following link: https://zenodo.org/record/5594600^13^, and in the Github repository https://github.com/covid19br/model-P1-variant.

### Nowcasting

Data for hospitalized COVID-19 cases among residents in Manaus from 01 November 2020 to 31 January 2021 was obtained from SIVEP-Gripe database of 15 February 2021. The hospitalized cases of the last 10 weeks in the time-series were nowcasted^14^ to correct for notification delay Data used in parameters estimation were collected from the SIVEP-Gripe In this system, reporting of cases can be delayed for several reasons, including the notification system itself and confirmation of RT-PCR test results. The nowcasting procedure estimates, based on the past delay distribution, the number of cases that already occurred but were not yet reported. A window of 10 weeks is the acting window on the series, since delays greater than this are rare.

Nowcasting requires a pair of dates: (i) onset date of the event and (ii) report date of the event. The delay distribution is modeled as being best described as a Poisson distribution for days since the onset date to the report date. We considered the first symptoms date as the onset date. For the report date, we used the latest between the test result date and the clinical classification date. The nowcasting algorithm were developed by ref. ^14^, and implemented in the *NobBS* (Nowcasting by Bayesian Smoothing) package in ${\mathsf{R}}$^15^.

### Model parametrization and initial condition estimation

The model requires appropriate mid-epidemic initial conditions in order to give relevant results. In the model, the number of new hospitalizations at a given time—*h*_*new*_, is directly proportional to the number of exposed individuals at that time, therefore data was used to get an approximation of the number of exposed people. Also, to quantify the number of people belonging to the recovered class, prevalence was used. In Table 1 we present the parameters considered for the wild-variant are described below. The parameters for the P.1 variant are the same except for those considered in the model fitting and in the Sensitivity Analysis 1 (SA1). See more detailed for the analysis of the initial conditions in the Supplementary Methods (Section 3 of the SI).

**Table 1:** Epidemiological parameters.

Parameter	Description	Value	Source
*γ*	Average time in days between being infected and developing symptoms	5.8	^25^
*ν*_*i*_	Average time in days between being infectious and recovering for asymptomatic and mild cases	9.0	^26^
*ν*_*s*_	Average time between being infectious and recovering/dying for severe cases	8.4	SIVEP-Gripe for São Paulo State
*ξ*	Reduction on the exposure of symptomatic cases (due to symptoms/quarantining)	0.1	Assumed
*ξ*_*sev*_	Reduction on the exposure of severe cases (due to hospitalization)	0.9	Assumed
*ω*	Relative infectiousness of pre-symptomatic individuals	1.0	Assumed
*α*	Proportion of asymptomatic cases	[0.67, 0.44, 0.31]	Juvenile^27^ Adult and Elderly^28^
*σ*	Proportion of symptomatic cases that require hospitalization	[0.001, 0.012, 0.089]^a^	^29^
*μ*	In-hospital mortality ratio	[0.417, 0.188, 0.754]	^11^
*χ*	Case report probability	1.0	Assumed

^a^The proportion is weighted by the age distribution of the population with each age category.

### Maximum likelihood estimation

Given the cumulative daily curves of hospitalization for wild-type variant (*C*_1_), and P.1 variant, (*C*_2_) we can obtain the daily variation of each curve (namely ${{\Delta }}{C}_{1}^{t}$ and ${{\Delta }}{C}_{2}^{t}$). Those curves are summed up to give the total number of weekly new cases:

1 $${{\Delta }}{C}^{\tau }=\mathop{\sum }\limits_{i=1}^{7}({{\Delta }}{C}_{1}^{\tau -1+i}+{{\Delta }}{C}_{2}^{\tau -1+i})$$

where *τ* is a discrete index given in weeks.

To calculate the frequency of P.1 in a given time period *T*, we use the proportion of new cases in this period from the wild-type and P.1 variant as follows:

2 $${P}^{t^{\prime} }=\frac{\mathop{\sum }\limits_{i=1}^{T}{{\Delta }}{C}_{2}^{T-1+i}}{\mathop{\sum }\limits_{i=1}^{T}{{\Delta }}{C}_{1}^{T-1+i}+\mathop{\sum }\limits_{i=1}^{T}{{\Delta }}{C}_{2}^{T-1+i}}$$

where $t^{\prime}$ is a discrete index given in *T* periods. The time period *T* depends on the dataset of genome sequences (weekly^3^ and monthly^12^).

Using the maximum likelihood method, we fitted the model by estimating five parameters, namely, the relative transmissibility (${{\Delta }}\beta =\frac{{\beta }_{2}}{{\beta }_{1}}$), the relative force of reinfection of P.1 (*p*_*r*_), initial total prevalence (*ρ*^0^ = [*R*/*N*]_*t*=0_), initial fraction of cases that were caused by the new variant (*P*^0^), and intrinsic growth rate of the wild-type variant (*r*). The initial fraction of P.1 cases (*P*^0^) accounts for the uncertainty in the time of emergence of the new variant: the simulation starts at beginning of November, so this initial value is below 1 individual, and only reaches this threshold by mid to late November, depending on the value of *P*^0^. The parameter *r* incorporates effects related to contact rates for the wild-type variant, such as non-pharmacological interventions relaxation, elections, and others; it affects the transmissibilities of both variants (*β*_1_ and *β*_2_) in the same way, and so is independent of Δ*β*.

Number of hospitalization cases were assumed to follow a Poisson distribution, with expected value given by Eq. (1). The recorded number of P.1 in genome samples was assumed to follow a binomial distribution with an expected value equal to the product of the total number of genome sequences sampled in each date and the proportion of P.1 cases (Eq. (2)). The log-likelihood function for the model fitting was then:

3 $${{{{{{{\mathcal{L}}}}}}}}=\mathop{\sum}\limits_{i}{{{{{{\mathrm{log}}}}}}}\,{{{{{{{\rm{Pois}}}}}}}}({x}^{i}| \lambda ={C}^{i})+\mathop{\sum}\limits_{j}{{{{{{\mathrm{log}}}}}}}\,{{{{{{{\rm{Bin}}}}}}}}({y}^{j}| N={n}^{j},\theta ({\pi }^{j})={P}^{j})\,,$$

where Pois is a Poisson distribution with parameter *λ*, *x*^*i*^ is the number of recorded hospitalizations in week *i*, Bin is a Binomial distribution with parameters *N* (total number of trials) and *π*^*j*^ (probability of success at each trial), *n*^*j*^ is total number of sequences in clinical samples in week or day *j*, *y*^*j*^ is the number of P.1 sequences in each of these samples, and *θ*(. ) is the *logit* function.

The model was then fitted by finding the values of the five above mentioned parameters that minimize the negative of the log-likelihood function (Eq. (3)), using the function mle2, from the R package *bbmle*^16^. To find starting values for the optimization performed by mle2 we calculated the log-likelihood function for one million combinations of parameters values in a regular reticulate within reasonable ranges. The 100 sets of parameters that were local minima (that is, with highest log-likelihood values) were used as starting values for the computational minimization.

The confidence intervals for the expected number of cases and frequency were estimated from 20,000 parametric bootstrap samples assuming that the estimated parameters follow a multivariate normal distribution. The parameters of these multivariate distributions were the estimated values and estimated variance-covariance matrix of the parameters. We then calculate the 2.5% and 97.5% quantiles of each parameter to obtain the confidence intervals of our estimates. We check the reliability of these estimates by verifying that the log-likelihood profiles satisfy required conditions for identifiability, as detailed in the Supplementary Methods (Section 4 of the SI and Fig. S1).

### Sensitivity analysis

The model fitting assumed a constant infection hospitalization rate (IHR, parameter *σ*) for each age group over time for both variants. Recent evidence suggests that prior SARS-CoV-2 infection protects most individuals against reinfection^17^, so reinfections might have lower IHR. Because the pathogenicity of the P.1 variant is unknown, the model fitting was repeated assuming that the odds ratio of the IHR in each age class for P.1 infections compared to wild-type variant infections (SA1) is a free parameter. Moreover, as the collapse of Manaus health system hindered hospitalizations of new severe cases and may have affected case recording in surveillance databases, the model fitting was repeated considering only the period prior to the collapse (10 January 2021) (SA2). Sensitivity analysis, latin hypercube explorations and likelihood profiles characterization are important methods which can be applied in this kind of model, where fitting depends on a set parameters, which were obtained from the literature and one has no further information about confidence intervals.

### Reporting summary

Further information on research design is available in the Nature Research Reporting Summary linked to this article.

## Results

**Fig. 2:**
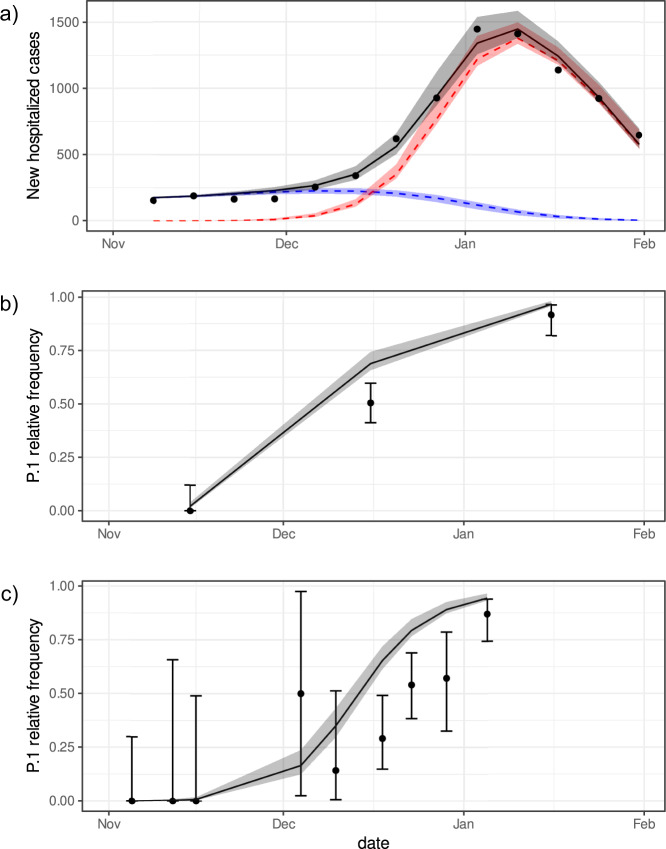
Hospitalization cases and frequency of the P.1 variant in Manaus city. **a** Weekly new hospitalized COVID-19 cases in Manaus city. Grey line represents the fitted values of total cases (all variants) by maximum likelihood estimation (MLE) of the parameters. Red and blue lines represent the predicted values of cases due to P.1 and wild-type variants, respectively. Black dots are nowcasted observed data of hospitalizations. Panels **b, c** show the fittings to the time-series frequency of P.1 on datasets provided by previous works, which were aggregated monthly^13^ and weekly^3^, respectively. The area around the lines indicate the 95% CI of the expected values. Dots and lines are the sample proportions of P.1 in sequenced genomes, and their 95% sample CI. The fitted values of the model parameters are presented in Table 2.

We present the fitted parameters on Table 2 and Fig. 2. The estimated transmissibility of P.1 was 2.6 (95% Confidence Interval (CI): 2.4–2.8) times higher compared to the wild-type variant, while the relative force of reinfection of the new variant was estimated to be 0.032 (CI: 0.026–0.040, Table 2). The fitted model also estimated that, at the time P.1 variant emerged, the prevalence of previous infection by the wild-type variant was 78% (CI: 73–83%), and that the number of cases by the wild-type variant were increasing with an estimated daily intrinsic growth rate of 0.029 days^−1^ (CI: 0.024–0.035 days^−1^). Given these parameter values, reinfections by P.1 accounted for 28% of the cases in Manaus from November 2020 through January 2021.

**Table 2:** Summary of the fitted parameters and respective confidence intervals considering the entire period, November 1, 2020 to January 31, 2021 maintaining the same pathogenicity of the previous variant.

Parameter	Main fitting			SA1			SA2		
	Estimate	2.5%	97.5%	estimate	2.5%	97.5%	Estimate	2.5%	97.5%
Relative transmission rate for the new variant	2.61	2.45	2.76	2.52	2.28	2.76	2.95	2.70	3.20
Relative force of reinfection of P.1	0.032	0.026	0.040	0.053	0.044	0.065	0.000	0.000	0.000
Prevalence of previous infection (2020-11-01) (%)	78	73	83	73	67	78	71	69	74
Initial fraction of the new variant (2020-11-01) (×10^−5^)	30.4	8.2	112.9	8.5	1.4	50.8	17.6	5.0	62.4
Intrinsic growth rate (days^−1^)	0.029	0.024	0.035	0.045	0.037	0.052	0.030	0.026	0.034
Relative IHR odds ratio	1^a^	–	–	0.74	0.63	0.85	1^a^	–	–

Sensitivity analyses were performed considering different pathogenicity of the P.1 variant (SA1) and data censuring after the collapse of the healthcare system (SA2) in Manaus, Brazil, on January 10, 2021.

^a^Parameter was fixed, not estimated, in this analysis.

We also evaluated the impact of a distinct pathogenicity of the P.1 variant on our estimates by allowing the infection hospitalization rate (IHR) of the new variant to be estimated as a free parameter (see SA1 in Table 2). The relative transmissibility and prevalence did not differ statistically from the the previous estimates and thus are robust to relaxing the assumption about equal pathogenicity for both variants. Oddly enough, the data gives no support for a higher IHR of P.1. Moreover, the model fit to hospitalization data prior to the healthcare system collapse in the city of Manaus (11 January, 2021) estimated an even larger transmissibility (SA2 in Table 2). We present additional results in the Supplementary Methods (Section 5 of the SI and Fig. S2), with plots of the number of individuals over time in each compartment (shown in Fig. 1) for both variants and the cumulative curves for hospitalized individuals using the main fitting parameters.

## Discussion

COVID-19 hospitalizations and frequency of the P.1 variant in clinical samples showed a sharp increase in Manaus, Brazil, starting December 2020. The fitted model describes this joint increase as the result of the emergence of P.1, estimated to be 2.6 times more transmissible than the wild-type variant. The spread of P.1 occurred despite a high estimated prevalence of infection by the wild-type virus both estimated (this present work) and empirically found^7,8^. The pathogenicity of P.1 is still unknown, but assuming hospitalization rates as a proxy for pathogenicity, the high P.1 transmissibility holds for different ranges of pathogenicity. Two recent studies analysed genomic data of SARS-CoV-2 from Manaus evaluating the transmissibility of the new variant^3,4^. Faria and collaborators integrated mortality and genomic data and, using a semi-mechanistic Bayesian model, estimated a transmissibility 1.4–2.2 times higher and 25–61% evasion of protective immunity related to the P.1 variant^3^. Naveca and collaborators estimated a 2.2 times higher effective reproduction number for the P.1 variant using phylogenetic methods, and suggested that P.1 is at least two times more transmissible than the parental lineage, assuming reinfections are rare^4^. The present work follows a different approach that can be defined as an epidemiological, model-based, and data-fitting approach, suitable for scenarios where only surveillance data are available, and applicable to other emerging variants throughout the world. Our epidemiological compartment model can provide point estimates of two key epidemiological parameters as soon as data on number of cases and frequency of a new variant are available. This is valuable for practical purposes, as estimates can be available timely for interventions and alerts. Also, we think we contribute for the field of mathematical models in epidemiology, showing a simple instance to link such class of models to data through maximum likelihood methods. Notably, all three different approaches estimated very high transmissibility of the P.1 variant.

Many knowledge gaps about the pandemic in the Amazonian region still remain. Population-based serological surveys are not available and thus prevalence was included in the fitted parameters. The analysed data overlapped with the period of the health system collapse. Aware that in-hospital fatality rates can quickly change when the health system is under stress^11^, we have chosen hospitalization data instead of mortality data (see subsection Dataset in Methods). Still, during the health system collapse many severe cases probably were not recorded in the system and remained unaccounted for. Our results were robust to removing this period in the sensitivity analysis (SA2). Even without P.1 emergence, the model estimates an increase in the number of cases (intrinsic growth rate parameter, see Table 2), which could be a consequence of loosening non-pharmacological interventions (NPIs)^9^, an effect of waning immunity, or both. Our model does not consider these effects explicitly, but by fitting the initial growth rate we indirectly account for their effects on the dynamics and on the estimation of the remaining parameters.

The impacts of a highly transmissible variant have already been highlighted by the spread of VOC B.1.1.7 in the UK, USA and Europe^18^. The variant B.1.1.7 has an upper-bound estimate for transmissibility of 2.3^5^, which is smaller than our lower bound estimate for P.1. Although P.1 is highly transmissible according to our model fitting, it has rarely been found at high frequencies in other countries outside of Latin America (but see Canada cases^19^ and CDC analysis showing P.1 variant grows in frequency in the United States of Amerca until June, 2021^20^). We believe that some factor may have contributed to the unsuccessfulness of P.1 variant across the world. First, we believe the warnings given by the Brazilian and Japanese authorities/researchers in January may have contained the spread of P.1, by travel ban increment to the restrictions already in place since 2019 for people who departed from Brazil or have stopped over in in the country before the P.1 emergence due to the uncontrolled epidemic situation in the country. Another important geographic aspect is that the main international airports linking Brazil to other countries are in the Southeast region (São Paulo and Rio de Janeiro), which reached high frequency of P.1 variant only after many travel bans were already in effect. Second, it should be expected that a variant of concern causing greater impact where it emerged and was naturally selected. Virus and the immune system are under a coevolutionary process which is usually composed by a geographic mosaic of coldspots and hotspots^21,22^. The previous variants circulating in Brazil, the environment and genetic conditions where P.1 emerged determines its success in Latin America, making this place a hotspot of the coevolution between the coronavirus and local populations. Such specific conditions might not be found in other places. Finally, it is also important to notice, for instance, that variants emerged in other continents (such as B.1.1.7 and B.1.351) have not caused the same damage in Brazil and other Latin-american countries as it caused in their place of emergence.

Notwithstanding the local emergence and likely greater concern for Latin America, P.1 has been raised to a *Variant of Concern* by experts of WHO and it remains in their official list up to today (together with B.1.1.7, B.1.351, and B.1.617) because of its potential related to the damage already caused and to the mutations it carries. Higher transmissibility of the P.1 variant raises strong concerns of swift upsurges in the number of cases once P.1 reaches community transmission. Although our estimate for the relative force of reinfection by the P.1 variant seems low, the impact is strong enough to drive, together with a high transmissibility, a large surge even in a population heavily affected by the wild-type variant. For instance, in Manaus, 28% of the new cases in the period considered were due to reinfections by P.1 in our estimations, which is in line with estimates from blood donors samples in Manaus city^23^. Relaxing the assumptiom of equal IHR for both variants highlighted that the IHR for P.1 may be lower, and then up to 40% of the new cases would be of reinfections (SA1). This lower value of IHR could be explained by the fact that more asymptomatic cases happen amongst reinfections compared to primary cases, as shown in the SIREN study^24^, and since our model does not consider differing proportions of asymptomatic cases between primary and reinfection cases, this led to overall reduction in IHR to the P.1 variant. However, in a scenario of low prevalence rate of infection by the wild-type variant, the high transmissibility is the most determinant parameter of the rapid increase in the number of cases and can lead to even sharper outbreaks. The P.1 variant has already been detected in at least 36 countries, with local transmission currently confirmed in five of them^18^. This points to the urgency of reinforcing measures to avoid a global spread of P.1, which include an agile global genomic surveillance network.

Further, to improve our ability to deal with the threat of P.1, it is urgent to study (i) the pathogenicity of the P.1 variant, since this trait, in association with high transmissibility, can drive even well-prepared health systems to collapse; (ii) the efficacy of current vaccines for P.1 variant infections; and iii) the main factors promoting the emergence of VOCs, specially the roles of previous high prevalence and of waning immunity.

## Supplementary information

Supplementary information

Reporting Summary

## Data Availability

The hospitalization data were obtained from the Brazilian epidemiological syndrome surveillance system for influenza, SIVEP-Gripe (https://opendatasus.saude.gov.br). Time-series of frequency of sequenced genomes identified as P.1 in Manaus were extracted from published datasets^3,12^. Data that support the findings of this study, including source data underlying the main figures in the manuscript, can be accessed via the link: https://zenodo.org/record/5594600^13^ or through the Github repository https://github.com/covid19br/model-P1-variant/tree/main/DATA.

## Code availability

The codes used for perform the analysis are available in the following link: https://zenodo.org/record/5594600^13^, and in the Github repository https://github.com/covid19br/model-P1-variant.
