# Supplementary material for: Model-based estimation of transmissibility and reinfection of SARS-CoV-2 P.1 variant
Source: Commun Med (Lond). 2021 Nov 15;1:48. doi: 10.1038/s43856-021-00048-6 (PMC9053218; doi:10.1038/s43856-021-00048-6)
Supplement: Supplementary file 1 — Supplementary information [file 43856_2021_48_MOESM1_ESM.pdf]

Supplementary Information for

Model-based estimation of transmissibility and reinfection of SARS-CoV-2 P.1 variant

Renato Mendes Coutinho, Flavia Maria Darcie Marquitti, Leonardo Souto Ferreira, Marcelo Eduardo Borges, Rafael Lopes Paixão da Silva, Otavio Canton, Tatiana P. Portella, Silas Poloni, Caroline Franco, Mateusz M. Plucinski, Fernanda C. Lessa, Antônio Augusto Moura da Silva, Roberto Andre Kraenkel, Maria Amélia de Sousa Mascena Veras and Paulo Inácio Prado

RMC, E-mail: renato.coutinho@ufabc.edu.br, and FMDM, E-mail: flamarquitti@gmail.com

This PDF file includes:

Supplementary Methods
Figs. S1 to S2
SI References

Supplementary Methods

Section 1 describes the model, section 2 describes the contact matrix used, section 3 describes the the choice of initial conditions, section 4 shows our exploration of the space for the estimated parameters, and finally in section 5 we show additional results with the curves estimated from our model for each compartment through the time window used here.

1. Model equations

The model is an extended Susceptible, Exposed, Infected, and Recovered (SEIR) model that comprises susceptible (S), pre-symptomatic (E), asymptomatic (A), mild symptomatic (I), severe/hospitalized (H), recovered (R) and deceased (D) compartments. These compartments are duplicated to account for a second variant of SARS-CoV-2, and each of them is stratified into three age classes: young (<20 years old), adults ($[20-59]$ years old), and the elderly (≥ 60 years old). Therefore, all the compartments (variables) and parameters are $\mathbb{R}^3$ elements. The "wild-type" classes represent all non-P.1 variants present, which do not seem to be variants of concern.

We assume that the second variant is capable of reinfecting individuals who have recovered from infection by the wild-type variant while the inverse is not possible; in the absence of data indicating this possibility, allowing reinfection by the wild-type variant on recovered of infection by P.1 would have negligible effect due to the small time window (3 months) considered in the present work. We also consider that a variant is not capable of reinfecting individuals recovered from the same lineage. Our model does not include vaccination due to low rates of vaccination in Brazil during the study time period.

To model the virus spread in the population, we assume that asymptomatic individuals have equal infectiousness compared to symptomatic ones, while pre-symptomatic individuals have reduced infectiousness represented by ω. To model behaviour, we assume that symptomatic individuals self-isolate themselves to some degree, reducing their contacts by ξ. Individuals with severe disease have greater isolation ξ_{sev} due to hospitalization. The daily contacts between each age class is represented by the matrix $\hat{\mathbf{C}}$ (see more information about the contact matrix in the next Section). The force of infection λ_k for each variant k is defined below:

$$\lambda_k = \beta_k \hat{\mathbf{C}}[A_k + \omega E_k + (1-\xi)I_k + (1-\xi_{sev})H_k]$$

The complete system of equations is given by:

Completely Susceptible

$$\frac{dS}{dt} = -\lambda_1 \frac{S}{N} - \lambda_2 \frac{S}{N} \tag{1a}$$

Wild variant

$$\frac{dE_1}{dt} = \lambda_1 \frac{S}{N} - \frac{E_1}{\gamma_1} \quad [1b]$$

$$\frac{dA_1}{dt} = \frac{(1-\sigma_1)\alpha_1 E_1}{\gamma_1} - \frac{A_1}{\nu_{i,1}} \quad [1c]$$

$$\frac{dI_1}{dt} = \frac{(1-\alpha_1)(1-\sigma_1)E_1}{\gamma_1} - \frac{I_1}{\nu_{i,1}} \quad [1d]$$

$$\frac{dH_1}{dt} = \frac{\sigma_1 E_1}{\gamma_1} - \frac{H_1}{\nu_{s,1}} \quad [1e]$$

$$\frac{dR_1}{dt} = \frac{A_1}{\nu_{i,1}} + \frac{I_1}{\nu_{i,1}} + \frac{(1-\mu_1)H_1}{\nu_{s,1}} - p_r \lambda_2 \frac{R_1}{N} \quad [1f]$$

$$\frac{dD_1}{dt} = \frac{\mu_1 H_1}{\nu_{s,1}} \quad [1g]$$

P.1 variant

$$\frac{dE_2}{dt} = \lambda_2 \frac{S}{N} - \frac{E_2}{\gamma_2} + p_r \lambda_2 \frac{R_1}{N} \quad [1h]$$

$$\frac{dA_2}{dt} = \frac{(1-\sigma_2)\alpha_2 E_2}{\gamma_2} - \frac{A_2}{\nu_{i,2}} \quad [1i]$$

$$\frac{dI_2}{dt} = \frac{(1-\alpha_2)(1-\sigma_2)E_2}{\gamma_2} - \frac{I_2}{\nu_{i,2}} \quad [1j]$$

$$\frac{dH_2}{dt} = \frac{\sigma_2 E_2}{\gamma_2} - \frac{H_2}{\nu_{s,2}} \quad [1k]$$

$$\frac{dR_2}{dt} = \frac{A_2}{\nu_{i,2}} + \frac{I_2}{\nu_{i,2}} + \frac{(1-\mu_2)H_2}{\nu_{s,2}} \quad [1l]$$

$$\frac{dD_2}{dt} = \frac{\mu_2 H_2}{\nu_{s,2}} \quad [1m]$$

Supplementary Equations

$$C_1(t) = \int_0^t \chi \sigma_1 \frac{E_1(t')}{\gamma_1} \mathrm{d}t' \quad [1n]$$

$$C_2(t) = \int_0^t \chi \sigma_2 \frac{E_2(t')}{\gamma_2} \mathrm{d}t' \ , \quad [1o]$$

where C_1 ad C_2 are the cumulative hospitalization cases reported, and each variable of the system ($S,\ E_k, ...,\ C_k$) is a vector containing each age class, *e.g.*, $E_1 = (E_{1,y}, E_{1,a}, E_{1,e})^T$. The equations were numerically solved by the R package developed by (1).

2. Contact Matrices

Our model includes three age group categories: young ($[0-19]\,y.o.$), adults ($[20-59]\,y.o.$), and elderly (greater than $60 y.o.$). To model contacts between these groups we use estimated contact

matrices computed by (2), but since the original matrices use five-year age bins going up to 95+ years, we aggregate classes leading to a 3×3 matrix in the following way:

Let A, B be sets of indexes forming age groups (not necessarily of equal sizes), $x_{i,j}$ denoting contact between age groups i and j in the original matrix, d_i denoting population size of the age group i. The new contact matrix $\hat{\mathbf{C}}$ is given by:

$$\hat{\mathbf{C}}_{A^*,B^*} = \frac{\sum_{i\in A}\sum_{j\in B} d_i x_{i,j}}{\sum_{i\in A} d_i} \qquad [2]$$

where A^*, B^* denotes a new indexation rule. Note that the contact matrices depend on local demographics and therefore must be computed for each place of study.

3. Initial Condition Estimation

The model requires appropriate mid-epidemic initial conditions in order to give relevant results. In the model, the number of new hospitalizations at a given time – h_{new}, is directly proportional to the number of exposed individuals at that time, therefore data was used to get an approximation of the number of exposed people. Also, to quantify the number of people belonging to the recovered class, seroprevalence was used.

We can estimate the appropriate initial conditions by finding an approximation for our model that relates more directly to the available data in each class. In the absence of the variant P.1, the model has four classes of infected compartments, namely $\mathbf{y} = (E_1,\ A_1,\ I_1,\ H_1)^T$, and another three classes, represented by $\mathbf{z}$, i.e., $\mathbf{z} = (S,\ R_1,\ D_1)^T$. To that effect, we can write the system as

$$\dot{\mathbf{y}} = F(\mathbf{y},\mathbf{z}) - G(\mathbf{y},\mathbf{z}), \qquad [3]$$

$$\dot{\mathbf{z}} = J(\mathbf{y},\mathbf{z}) \ , \qquad [4]$$

where F comprises all entries of new Infected, coming from classes $\mathbf{z}$, whilst G accounts for the transitions within infected classes and also recovery and death from the disease. Then, to find a good approximation for a small time window, we perform a linearization of our model around a point $(\mathbf{y},\mathbf{z})$. Keeping $\mathbf{z}$ fixed, we get

$$\dot{\mathbf{y}} = (\hat{F} - \hat{G})\mathbf{y} \ , \qquad [5]$$

where $\hat{F}$ and $\hat{G}$ are the linearized matrices arising from the functions F and G, respectively. The only entrance of new infected comes from the $\beta S\lambda/N$ terms in the $\dot{E}_1 = (\dot{E}_{1,y}, \dot{E}_{1,a}, \dot{E}_{1,e})^T$ equations (sub-indexes are y young, a adults and e elderly), then, the only non-zero elements of $\hat{F}$ are in its first 3 lines. Before proceeding, it is useful to define

$$\hat{b} = \mathrm{diag}(S)\hat{\mathbf{C}} \qquad [6]$$

which allow us to write

$$\hat{F} = \frac{\beta}{N}\begin{bmatrix} \omega\hat{b} & \hat{b} & (1-\xi)\hat{b} & (1-\xi_{sev})\hat{b} \\ & & \mathbb{0}_{9,12} & \end{bmatrix} \qquad [7]$$

$\hat{G}$ contains the terms of Exposed, E_1, the 3 possible forms of the disease considered in the model $(A_1, I_1$ and $H_1)$, as the terms in its first 3 rows, whilst the remainder of its main diagonal contains

terms of recovery and death. For simplicity, every constant (or vector for the terms with σ) in $\hat{G}$ expression Eq. (8) should be thought as diagonal matrices with its elements given by the constants (or vectors) and every $\mathbb{0}$ is a 3-dimensional square matrix where all entries are null.

$$\hat{G} = \begin{bmatrix} \gamma^{-1} & \mathbb{0} & \mathbb{0} & \mathbb{0} \\ -\alpha(1-\sigma)\gamma^{-1} & \nu_i^{-1} & \mathbb{0} & \mathbb{0} \\ -(1-\alpha)(1-\sigma)\gamma^{-1} & \mathbb{0} & \nu_i^{-1} & \mathbb{0} \\ -\sigma\gamma^{-1} & \mathbb{0} & \mathbb{0} & \nu_s^{-1} \end{bmatrix} \qquad [8]$$

The linearization above implies that, for a small time interval, $\mathbf{y}$ has an exponential behavior and that the eigenvalues of $\hat{L} = \hat{F} - \hat{G}$ are related to the exponential growth rates. Therefore, a short time after the beginning of the epidemic, the largest eigenvalue should be the one to dominate. So the exponential growth rate of the wild-type variant – r, can be matched to the largest eigenvalue of $\hat{L}$ to obtain an estimate for β. The eigenvector associated with the largest eigenvalue gives the proportions of infected classes, which, together with the estimated number of exposed individuals – $E_1 = \gamma_1 h_{new}/\sigma_1$, results in an approximation for the number of people in the other infected classes.

Given a β, the largest eigenvalue of the linearization matrix is computed using the `eigs` function of the R package *rARPACK* (3) and we find the β that gives r as the largest eigenvalue through bisection root finding. Finally, subtracting the number of recovered and infected from the total population gives the number of susceptible individuals.

4. Identifiability and estimation of parameters' confidence intervals

We have systematically explored the log-likelihood surface by calculating it across an orthogonal grid of parameter values, and from that we built the log-likelihood profiles of the fitted parameters (Fig. S1). These are obtained by taking, for each parameter value along the x-axis in each plot, the minimum negative log-likelihood found across all other parameters with that value fixed. We initially varied all parameters in a regular grid with a rough resolution (yielding Fig. S1, left), over a wide range of parameter values. For each parameter combination in the grid we thus calculated the value of the negative log-likelihood function of the model. In all cases, the profiles showed a clear unique minimum, which is consistent with a global minimum in the parameter space we investigated. As detailed in the main text (Methods), we used the 100 points in the grid with lowest negative likelihood as starting values to numeric optimization routines (4, 5), to obtain the maximum likelihood estimates (MLEs) of the model parameters. All those points were then in the region of global minimum. Such numeric routines also provided the values of the negative log-likelihood at the vicinity of the MLEs (Fig. S1, right). These profiles were smooth and parabolic enough to assume that our estimates are identifiable, and also to allow an approximation of the distribution of the estimates by a multivariate Gaussian distribution, which we used to estimate confidence intervals of the estimated values of the parameters.

5. Full model solutions

In figure S2 we show the number of individuals in each class from both "wild" and the VOC P.1 weekly over the time we evaluated our model (November-1, 2020–February-28, 2021). The numerical results were performed with the main fitting parameters found by the estimation method (see Table 1 in the main text). All the fixed parameters used are available in Table 2 of the main text.

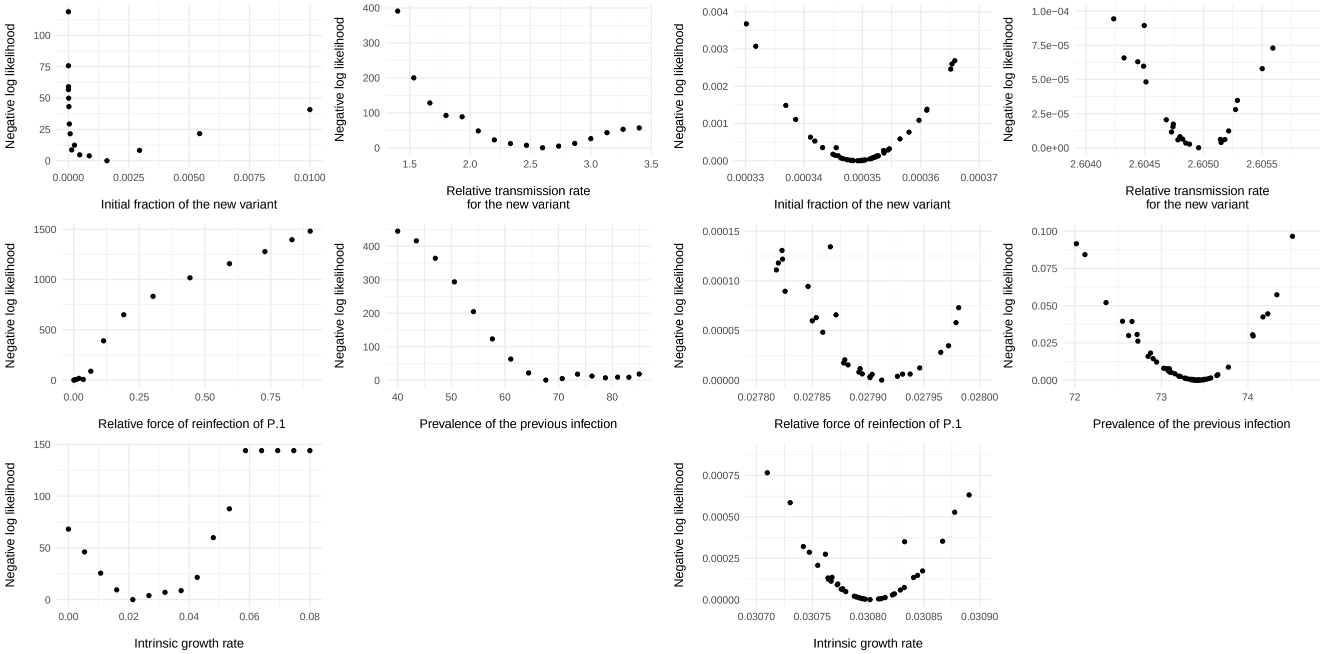

Fig. S1. Negative log-likelihood profiles for the fitted parameters (main fit). **Left**: global analysis, investigating the whole parameter ranges, but with low resolution. **Right**: high resolution profile around the global minimum.

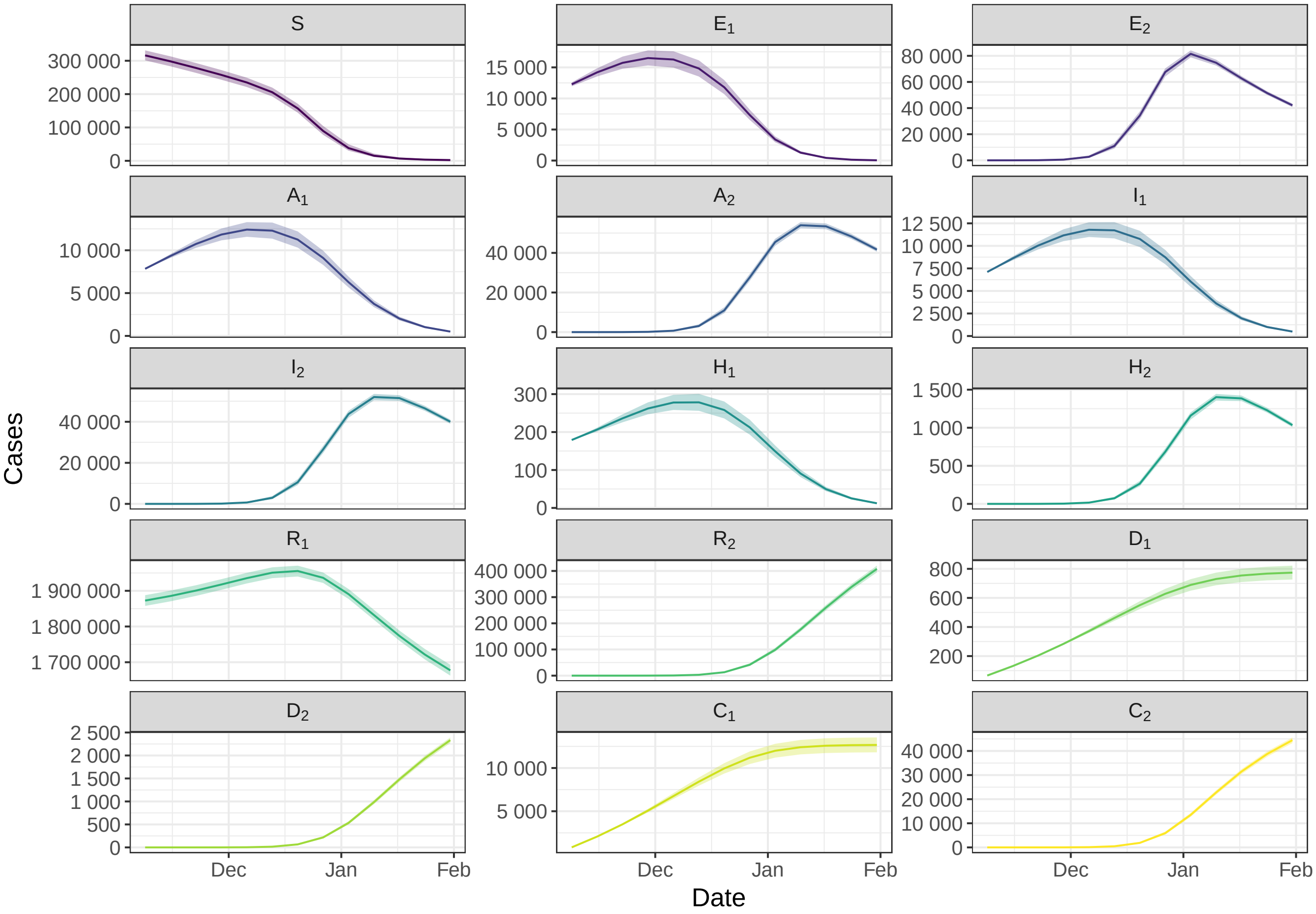

Fig. S2. In the y-axis we present the number of individuals in each compartment for "wild" (sub-index 1) and the VOC P.1 (sub-index 2) from Nov-1, 2020–Feb-28, 2021) computed at the end of the epidemiological weeks. The shaded area represents 95% confidence interval according to 1,000 bootstrap simulations of the parameters estimated by the main fitting. The compartments are: S: Susceptible, E: Exposed (pre-symptomatic), H: Hospitalized (severe infected individuals), I: Infected (symptomatic individuals, not hospitalized), A: Asymptomatic. D: Deceased, R: Recovered. For this result, we added all the 3 age categories. C_1 and C_2 are the cumulative number of hospitalizations for the two variants.

Supplementary References

1. K Soetaert, T Petzoldt, RW Setzer, Solving differential equations in R: Package deSolve. *J. Stat. Softw.* **33**, 1–25 (2010).
2. K Prem, et al., Projecting contact matrices in 177 geographical regions: an update and comparison with empirical data for the covid-19 era. *medRxiv* (2020).
3. Y Qiu, J Mei, MY Qiu, *r'ARPACK': Solvers for Large Scale Eigenvalue and SVD Problems*, (2016) R package version 0.11-0.
4. B Bolker, R Development Core Team, *bbmle: Tools for General Maximum Likelihood Estimation*, (2020) R package version 1.0.23.1.
5. JC Nash, R Varadhan, G Grothendieck, *optimx: Expanded Replacement and Extension of the 'optim Function*, (2021) R package version 2021-6.12.
