# Supplementary material for: Model-based estimation of transmissibility and reinfection of SARS-CoV-2 P.1 variant
Source: Commun Med (Lond). 2021 Nov 15;1:48. doi: 10.1038/s43856-021-00048-6 (PMC9053218; doi:10.1038/s43856-021-00048-6)
Supplement: Supplementary file 2 — Reporting Summary [file 43856_2021_48_MOESM2_ESM.pdf]

nature research

Corresponding author(s): DBPR COMMSMED-21-0205-T

Last updated by author(s): Oct 25, 2021

Reporting Summary

Nature Research wishes to improve the reproducibility of the work that we publish. This form provides structure for consistency and transparency in reporting. For further information on Nature Research policies, see our Editorial Policies and the Editorial Policy Checklist.

Statistics

For all statistical analyses, confirm that the following items are present in the figure legend, table legend, main text, or Methods section.

n/a	Confirmed	
☒	☐	The exact sample size (*n*) for each experimental group/condition, given as a discrete number and unit of measurement
☒	☐	A statement on whether measurements were taken from distinct samples or whether the same sample was measured repeatedly
☒	☐	The statistical test(s) used AND whether they are one- or two-sided<br>*Only common tests should be described solely by name; describe more complex techniques in the Methods section.*
☒	☐	A description of all covariates tested
☐	☒	A description of any assumptions or corrections, such as tests of normality and adjustment for multiple comparisons
☐	☒	A full description of the statistical parameters including central tendency (e.g. means) or other basic estimates (e.g. regression coefficient) AND variation (e.g. standard deviation) or associated estimates of uncertainty (e.g. confidence intervals)
☒	☐	For null hypothesis testing, the test statistic (e.g. *F*, *t*, *r*) with confidence intervals, effect sizes, degrees of freedom and *P* value noted<br>*Give P values as exact values whenever suitable.*
☒	☐	For Bayesian analysis, information on the choice of priors and Markov chain Monte Carlo settings
☒	☐	For hierarchical and complex designs, identification of the appropriate level for tests and full reporting of outcomes
☒	☐	Estimates of effect sizes (e.g. Cohen's *d*, Pearson's *r*), indicating how they were calculated

Our web collection on statistics for biologists contains articles on many of the points above.

Software and code

Policy information about availability of computer code

Data collection	Data used in this work are from the anonymized and public available data of the Brazilian national health surveillance of hospitalized individuals, system designed for the epidemiological syndrome for influenza (https://opendatasus.saude.gov.br), called SIVEP-Gripe. We filtered data for Manaus city and confirmed COVID19 cases, considering the period between November-1, 2020 and January-31, 2021. The codes used for perform the analysis are available in the Github repository \url{https://github.com/covid19br/model-P1-variant}<br><br>Time-series of frequency of sequenced genomes identified as P.1 in Manaus were extracted from published datasets:<br>1) Rede Genômica Fiocruz, Plots of lineages presence by state (2021) http://www.genomahcov.fiocruz.br/presenca-das-linhagens-por-estado, Accessed on 2021-02-28.<br>2) NR Faria, et al., Genomics and epidemiology of a novel sars-cov-2 lineage in Manaus , Brazil. MedRxiv , 1–44 (2021).
Data analysis	We used R for performing the analysis of fitting. The codes used for perform the analysis are available in the Github repository: https://github.com/covid19br/model-P1-variant

For manuscripts utilizing custom algorithms or software that are central to the research but not yet described in published literature, software must be made available to editors and reviewers. We strongly encourage code deposition in a community repository (e.g. GitHub). See the Nature Research guidelines for submitting code & software for further information.

Data

Policy information about availability of data

All manuscripts must include a data availability statement. This statement should provide the following information, where applicable:

- Accession codes, unique identifiers, or web links for publicly available datasets
- A list of figures that have associated raw data
- A description of any restrictions on data availability

The hospitalization data were obtained from the Brazilian epidemiological syndrome surveillance system for influenza, SIVEP-Gripe (\url{https://opendatasus.saude.gov.br}). Time-series of frequency of sequenced genomes identified as P.1 in Manaus were extracted from published datasets (Faria et al 2021, Rede Genômica Fiocruz 2021). Data that support the findings of this study, including source data underlying the main figures in the manuscript, can be accessed via the link: https://zenodo.org/record/5594600 (Coutinho et al 2021) or through the Github repository https://github.com/covid19br/model-P1-variant/tree/main/DATA.

Field-specific reporting

Please select the one below that is the best fit for your research. If you are not sure, read the appropriate sections before making your selection.

☒ Life sciences ☐ Behavioural & social sciences ☐ Ecological, evolutionary & environmental sciences

For a reference copy of the document with all sections, see nature.com/documents/nr-reporting-summary-flat.pdf

Life sciences study design

All studies must disclose on these points even when the disclosure is negative.

Sample size	We worked with the complete available data of hospitalized COVID confirmed cases for Manaus city considering the period between November-1, 2020 and January-31, 2021
Data exclusions	No exclusions were made.
Replication	No replication was made because we worked with the full available time-series data.
Randomization	Not relevant for this study.
Blinding	Bliding is not relevant for this study because we worked with the full available time-series data.

Reporting for specific materials, systems and methods

We require information from authors about some types of materials, experimental systems and methods used in many studies. Here, indicate whether each material, system or method listed is relevant to your study. If you are not sure if a list item applies to your research, read the appropriate section before selecting a response.

Materials & experimental systems

n/a	Involved in the study	
☒	☐	Antibodies
☒	☐	Eukaryotic cell lines
☒	☐	Palaeontology and archaeology
☒	☐	Animals and other organisms
☒	☐	Human research participants
☒	☐	Clinical data
☒	☐	Dual use research of concern

Methods

n/a	Involved in the study	
☒	☐	ChIP-seq
☒	☐	Flow cytometry
☒	☐	MRI-based neuroimaging
